# Modified Needle-Tip PcrV Proteins Reveal Distinct Phenotypes Relevant to the Control of Type III Secretion and Intoxication by *Pseudomonas aeruginosa*

**DOI:** 10.1371/journal.pone.0018356

**Published:** 2011-03-29

**Authors:** Hiromi Sato, Meredith L. Hunt, Joshua J. Weiner, Andrew T. Hansen, Dara W. Frank

**Affiliations:** Center for Infectious Disease Research, Department of Microbiology and Molecular Genetics, Medical College of Wisconsin, Milwaukee, Wisconsin, United States of America; University of California Merced, United States of America

## Abstract

The type III secretion system (T3SS) is employed to deliver effector proteins to the cytosol of eukaryotic hosts by multiple species of Gram-negative bacteria, including *Pseudomonas aeruginosa*. Translocation of effectors is dependent on the proteins encoded by the *pcrGVHpopBD* operon. These proteins form a T3S translocator complex, composed of a needle-tip complex (PcrV), translocons (PopB and PopD), and chaperones (PcrG and PcrH). PcrV mediates the folding and insertion of PopB/PopD in host plasmic membranes, where assembled translocons form a translocation channel. Assembly of this complex and delivery of effectors through this machinery is tightly controlled by PcrV, yet the multifunctional aspects of this molecule have not been defined. In addition, PcrV is a protective antigen for *P. aeruginosa* infection as is the ortholog, LcrV, for *Yersinia*. We constructed PcrV derivatives containing in-frame linker insertions and site-specific mutations. The expression of these derivatives was regulated by a T3S-specific promoter in a *pcrV*-null mutant of PA103. Nine derivatives disrupted the regulation of effector secretion and constitutively released an effector protein into growth medium. Three of these regulatory mutants, in which the linker was inserted in the N-terminal globular domain, were competent for the translocation of a cytotoxin, ExoU, into eukaryotic host cells. We also isolated strains expressing a delayed-toxicity phenotype, which secrete translocators slowly despite the normal level of effector secretion. Most of the cytotoxic translocation-competent strains retained the protective epitope of PcrV derivatives, and Mab166 was able to protect erythrocytes during infection with these strains. The use of defined PcrV derivatives possessing distinct phenotypes may lead to a better understanding of the functional aspects of T3 needle-tip proteins and the development of therapeutic agents or vaccines targeting T3SS-mediated intoxication.

## Introduction

*Pseudomonas aeruginosa* is a Gram-negative opportunistic pathogen responsible for severe nosocomial pneumonias, acute infections of immunocompromised individuals, and chronic infections of cystic fibrosis patients [1], [2]. Morbidity and mortality is due to a combination of host factors, the severity of tissue injury, and the intrinsic resistance displayed by *P. aeruginosa* to many therapeutic drugs [3], [4]. A major virulence factor used by *P. aeruginosa* during infection is the type III secretion system (T3SS) [2], [5]–[7]. Many Gram-negative pathogens employ T3SSs, which can be likened to a molecular syringe, to inject toxins directly into eukaryotic cells [8], [9]. Four known *P. aeruginosa* T3SS toxins, ExoS, ExoT, ExoU, and ExoY, have been identified to date. ExoS and ExoT interfere with eukaryotic cell signaling pathways and host cytoskeletal architecture by their bifunctional Rho GAP and ADP-ribosyltransferase activities [10]–[13]. ExoU functions as a phospholipase A_2_ [14]. ExoY is an adenylyl cyclase that shares homology to the edema factor of the anthrax toxin [15]. Of the four identified effectors, ExoU and ExoS are cytotoxic. The intracellular delivery of these enzymes and their interaction with eukaryotic cofactors is highly correlated with the dissemination of bacteria from the initial sites of infection and the induction of sepsis [5], [16]–[18].

The *P. aeruginosa* T3SS components (Psc) belong to the family of proteins that include orthologs encoded by *Yersinia* (Ysc) and by *Aeromonas* (Asc) [19]–[21]. Intoxication of mammalian cells by *P. aeruginosa* requires the products of the *pcrGVHpopBD* operon [7], [16], [22]. PcrV, PopB, and PopD are classified as translocators and mediate the injection of the effectors into eukaryotic cells or contact-dependent lysis of erythrocytes [23]–[26]. PopB and PopD are hydrophobic proteins that interact with or insert into membrane lipids, forming a channel called the translocon [22], [24], [27]. The translocon assembly is necessary for delivery of T3S effectors across the eukaryotic plasma membrane into the cytosolic compartment (reviewed in [8], [9], [28]). PcrV is a hydrophilic protein forming the T3-needle tip complex, which is required for appropriate assembly and insertion of PopB and PopD into host membranes [24], [27]. Strains carrying a *pcrV*-null mutation are noncytotoxic due to the inability to assemble the translocon [24], [29]. Complementation with a wild-type copy of the gene restores type III translocation and cytotoxicity. These results suggest that PcrV is involved in the regulation of effector translocation [29].

Of these translocator proteins only PcrV is an important protective antigen against T3SS-mediated *Pseudomonas* infection [29]–[31]. Active immunization with recombinant PcrV protects mice from lethal infection even under induced-leukocytopenia conditions [29], [32]. Passive protection against cellular intoxication, lung injury, and bacteremia due to *P. aeruginosa* infection in animal models was demonstrated with polyclonal antiserum or affinity-purified antibodies, a monoclonal antibody (Mab166), and a humanized F(ab′)_2_ single chain antibody against PcrV [29], [30], [32], [33]. These data provide further evidence for the critical role of PcrV in translocation events and suggest that PcrV is exposed on the bacterial surface rendering it accessible to antibody neutralization.

In this study we used an unbiased genetic approach to further define the functional domains of PcrV based on phenotypic analyses. A transposon-based system was used to randomly insert an in-frame linker into PcrV, and the derivatives were expressed under T3SS regulation in *pcrV*-deleted PA103. Based on phenotypic analyses, we grouped the linker-insertion and site-specific mutants into 4 classes. Each class of mutants exhibited different functional properties, suggesting that these derivative molecules will be useful towards understanding the multifunctional aspects of T3S tip proteins.

## Results

### Protein structure modeling of PcrV

**Figure 1:**
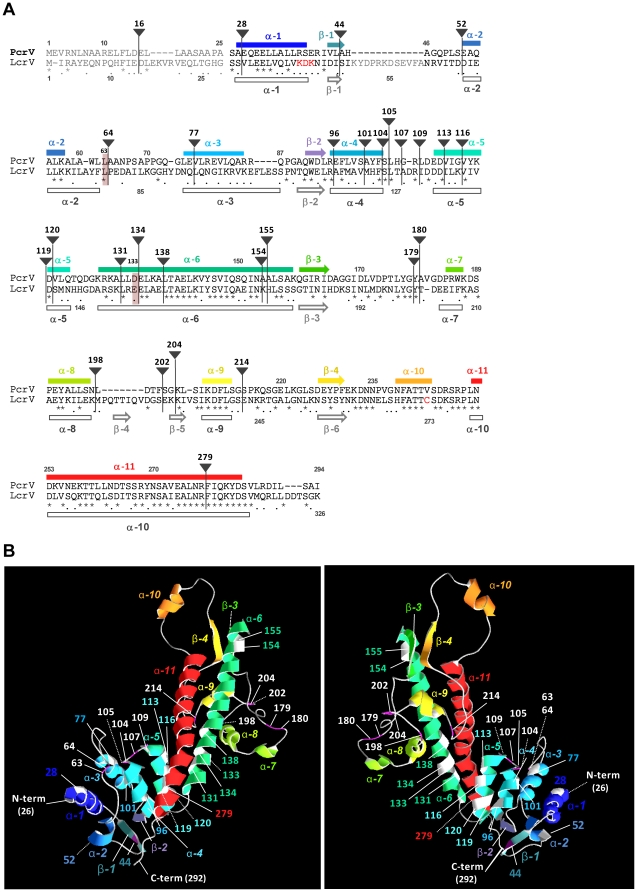
Location of the EZ-linker insertions in PcrV. (A) Alignment of amino acid sequences and predicted secondary structures of PcrV and *Y. pestis* LcrV. Linker insertion sites are indicated as a triangle tag with a residue number. Site-specific point mutations are indicated with red shaded bars. Colors of predicted secondary structures (α-helix, bar; β-sheet, arrow) in PcrV correspond to the colors in the 3D models in (B). Secondary structures of LcrV are shown below the alignment (gray). N-terminal regions in the LcrV crystal structure possessing no interpretable electron density are indicated in light gray. For crystallization of LcrV, charged residues at 40 to 42 and a cysteine residue at 273 (shown in red) were replaced with alanine and serine residues, respectively. (B) Location of linker insertions in the tertiary structure models of wild-type PcrV. For modeling, LcrV (1r6f chainA) was used as a template in Swiss Model. Predicted secondary structures are colored in a succession mode. Location of linker insertions is shown as white highlights in α-helices and magenta shading in β-sheets and loops. Left versus right panels: front and back views of PcrV (rotated 180°).

Recent progress using electron microscopy, molecular genetics, and structural biology approaches has increased our understanding of the mechanistic aspects of the T3SS translocation machinery. Visualization of the Ysc family members of needle-tip complexes by scanning transmission electron microscopy demonstrated that these molecules consist of three structural domains called the head, neck, and base [34]. The crystal structure of LcrV from *Yersinia* indicates that the protein has an overall dumbbell shape with a “grip” formed by the antiparallel coiled-coil of two long α-helices and globular domains at each end [35]. When the amino-acid sequences of *Y. pestis* LcrV and PcrV from several *P. aeruginosa* strains were aligned, the proteins possess 37 to 41% identity (Fig. 1A) and have sufficient similarity to produce superimposable structural models, with the model for PcrV shown in Fig. 1B (Swiss Model server, http://www.expasy.org) [36]. The LcrV structure information in this study, modeled from the data deposited in PBD (1r6f chain A), demonstrated subtle differences from the original crystallography study by Derewanda *et al.* [35]. The newer version of the modeling program (Swiss-protein, 2008) did not assign the previously predicted two α-helices, α3 and α8, in LcrV, thus the numerical nomenclatures of helices in this study are not identical to the original model.

Broz *et al.* determined that the N-terminal globular domain of three Ysc-family tip proteins, LcrV, PcrV, and AcrV (*Aeromonas*), forms the base of the tip complex that faces the *Yersinia* needle protein, YscF [34]. Based on the tertiary structure models, the base structure of PcrV corresponds to the N-terminal α-helices and β-sheets, α1, β1, α2, α3, β2, α4, and α5 (Fig. 1A and B). The C-terminal globular domain of LcrV forms the head of the tip complex and the neck is presumably the intramolecular coiled-coil region [34]. LcrV and PcrV contain a high structural similarity in the long coiled-coil region, which is a neck structure formed by α6 and α11 of PcrV (Fig. 1B). The C-terminal globular domain of PcrV is composed of several short α-helices and β-sheets (β3, α7, α8, α9, β4, and α10) with loop structures between (Fig. 1A and B).

### Scanning linker mutagenesis of PcrV

To understand the structure-function relationships of the multifunctional protein PcrV, we used a genetic approach to randomly introduce a 57-bp linker throughout the molecule. The plasmid containing *pcrV* with a type III promoter for *exoS*, pUCP-pS-*pcrV* (amp^r^), was mutagenized using an *in vitro* transposition system, which results in the insertion of a kanamycin marker (kan^r^) flanked by NotI sites. Approximately 129 plasmids that expressed both kan^r^ and amp^r^ in *E. coli* were screened by restriction mapping to identify the insertion within the *pcrV* coding region. Removal of the kan^r^ cassette by digestion with NotI and religation leaves 57 base pairs, resulting in an in-frame insertion of the 19-amino-acid EZ linker. Twenty-eight distinct in-frame insertion mutants were isolated. Derivatives are designated as EZ followed by the amino acid location where the linker was inserted or where the original PcrV amino-acid residue was substituted with another residue (Table 1 and 2). Insertions were localized within the predicted alpha helices of PcrV, α1, α3, α4, α5, α6 and α11 as well as in regions flanking these structural features (Fig. 1A and B). The pUCP-pS-*pcrV* plasmids carrying a linker insertion were transformed into a *pcrV*-null strain of *P. aeruginosa*, PA103Δ*pcrV*, and expression of the PcrV::EZ proteins was assessed by Western blot analysis with polyclonal anti-PcrV IgG. Compared to PcrV expressed from a complementation construct, derivatives had a slightly altered mobility in SDS-PAGE due to the additional 19 residues and the various amino acid sequences encoded within the insertion (Table 2). The sequence within the linkers differed by the target site duplication and three possible open reading frames based on the site of transposition (insertion frames are shown in Table 2).

**Table 1:** *Pseudomonas aeruginosa* strains used in this study.

Strain	Relevant characteristics/description
PA103	Wild-type cytotoxic strain, expresses type III proteins and translocates effectors, ExoU and ExoT.
PA103+pUCP-*pS-pcrV*	Wild-type strain harboring a plasmid copy of *pcrV* under control of the type III *exoS* promoter. Cytotoxic.
PA103*exsA*Ω+pUCP	Type III knockout strain, mutation in the type III regulatory gene, *exsA*, prevents activation of the type III secretion system. Noncytotoxic.
PA103Δ*pcrV*+pUCP (PA103ΔV+pUCP)	Nonpolar *pcrV* deletion harboring a plasmid. Noncytotoxic and constitutive secretion.
PA103Δ*pcrV*+pUCP-*pS-pcrV*(PA103ΔV+pcrV)	Complemented mutant harboring a plasmid copy of *pcrV* under control of the type III *exoS* promoter. Cytotoxic.
PA103ΔV+pUCP-*pS-exoUS142A*(PA103ΔV+*exoU*-S142A)	Nonpolar *pcrV* mutant harboring a plasmid copy of *exoU* with a point mutation at its catalytic site. Noncytotoxic and constitutive secretion.
PA103Δ*popB* (PA103ΔB)	Nonpolar *popB* deletion. Noncytotoxic.
PA103Δ*popB*+pUCP-*pS-popB*(PA103ΔB+*popB*)	Complemented mutant harboring a plasmid copy of *popB*. Cytotoxic.
PA103ΔUT+pUCP(PA103ΔUT+pUCP)	ExoU and ExoT knockout strain harboring a plasmid. Regulated secretion and noncytotoxic.
PA103ΔUT+pUCP-*pS-exoUS142A*(PA103ΔUT+*exoU*-S142A)	ΔUT strain harboring a plasmid copy of *exoU* with a point mutation at its catalytic site. Regulated secretion and noncytotoxic.
PA103Δ*pcrV*+pUCP-*pS*-*pcrV-*EZ(PA103ΔV+EZ)EZ16, EZ28, EZ77, EZ96, EZ101, EZ104, EZ105, EZ107, EZ109, EZ113, EZ116, EZ154, EZ155, EZ179, EZ180, EZ198, EZ202, EZ204, EZ214	Nonpolar *pcrV* mutant harboring a plasmid copy of *pcrV*::EZ linker.Regulated secretion and cytotoxic.
PA103ΔV+EZ44, EZ52, EZ64	Deregulated secretion and cytotoxic.
PA103ΔV+EZ119, EZ120, EZ131, EZ134, EZ138, EZ279	Deregulated secretion and noncytotoxic.
PA103Δ*pcrV*+pUCP-*pS*-*pcrV-*L63A or D133A (PA103ΔV+L63A or D133A)	Nonpolar *pcrV* mutant harboring a plasmid copy of *pcrV* with a point mutation at amino acid residue 63 or 133. Regulated secretion with delayed cytotoxicity.
PA103+pUCP-*pS*-*pcrV*-EZ (PA103+EZ) EZ44, EZ52, EZ64, EZ119, EZ120, EZ131, EZ134, EZ138, EZ279	Wild-type strain harboring a plasmid copy of pcrV::EZ-linker.Regulated secretion and cytotoxic.
PA103+pUCP-*pS*-*pcrV*-L63A or D133A(PA103+L63A or D133A)	Wild-type strain harboring a plasmid copy of pcrV with a point mutation at amino acid residue 63 or 133. Regulated secretion and cytotoxic.

**Table 2:** EZ-linker insertion sites and inserted amino acid residues.

mutation site	Insertion mapped to amino acid	insertion frame	inserted amino acid residues
EZ 16	15	1	CLLYTSCGRKMCTRDRFLD
EZ 28	27	1	CLLYTSCGRKMCTRDSASA
EZ 44	43	1	CLLYTSCGRKMCTRDRIVL
EZ 52	52	1	DCLLYTSCGRKMCTRDRLS
EZ 64	63	2	SLVHILRPQDVYKRQAWLL
EZ 77	77	3	AVSCTHLAAARCVQETGLE
EZ 96	96	2	LSLVHILRPQDVYKRQDLR
EZ 101	100	3	VSCTHLAAARCVQETVLVS
EZ 104	104	2	LSLVHILRPQDVYKRQAYF
EZ 105	105	3	PVSCTHLAAARCVQETDFS
EZ 107	107	3	AVSCTHLAAARCVQETGLH
EZ 109	108	1	CLLYTSCGRKMCTRDRHGR
EZ 113	112	1	CLLYTSCGRKMCTRDSDED
EZ 116	115	1	CLLYTSCGRKMCTRDSVIG
EZ 119	119	2	LSLVHILRPQDVYKRQVYK
EZ 120	119	1	CLLYTSCGRKMCTRDSYKD
EZ 131	130	1	CLLYTSCGRKMCTRDRRKA
EZ 134	134	1	DCLLYTSCGRKMCTRDRLD
EZ 138	137	3	VSCTHLAAARCVQETELKA
EZ 154	154	2	LSLVHILRPQDVYKRQQIN
EZ 155	154	1	CLLYTSCGRKMCTRDRINA
EZ 179	179	2	LSLVHILRPQDVYKRQLYG
EZ 180	179	1	CLLYTSCGRKMCTRDRYGY
EZ 198	197	1	CLLYTSCGRKMCTRDRLSN
EZ 202	201	1	CLLYTSCGRKMCTRDRDTF
EZ 204	203	1	CLLYTSCGRKMCTRDSFSG
EZ 214	213	1	CLLYTSCGRKMCTRDSLSG
EZ 279	278	1	CLLYTSCGRKMCTRDRLNR

### Cytotoxicity and secretory-regulation phenotypes of PcrV::EZ expressed in a *pcrV*-null strain

**Figure 2:**
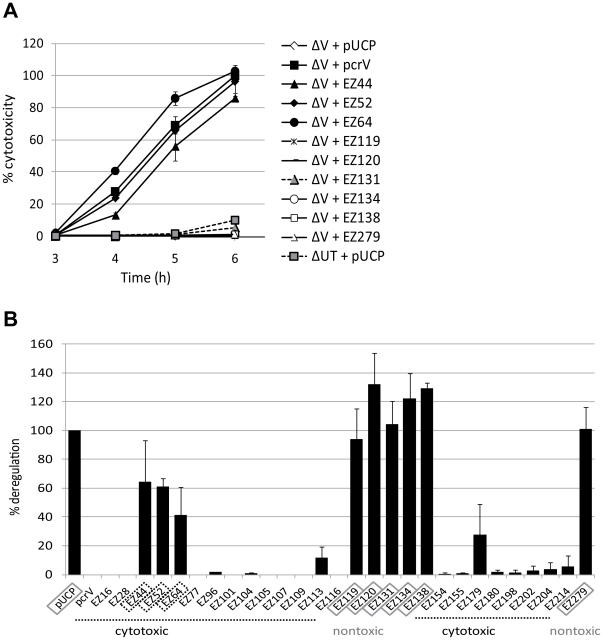
Cytotoxicity and secretion profiles of PA103Δ*pcrV* host strain complemented with *pcrV*::EZ-linker constructs. (A) LDH release from HeLa cells as a quantitative measurement of cytotoxicity during infection. Cell culture supernatants were assayed for LDH activity in triplicate of two independent experiments for statistical analyses. Representative strains are shown. Error bars indicate SD. (B) Secretory regulation profiles of PcrV derivatives. Bacterial cells were grown to suppress (−NTA) type III secretion and culture supernatants were subjected to Western blot analyses to quantify ExoU release. Deregulated ExoU secretion (highlighted by boxes) was quantified based on the amount of constitutively secreted ExoU by PA103Δ*pcrV* vector control (pUCP) as 100%. Deregulated/cytotoxic and deregulated/noncytotoxic phenotypes were shown as dotted and gray boxes, respectively. Results are representative of at least three independent experiments.

In cell culture systems, a *pcrV*-deletion strain (PA103Δ*pcrV)* releases effector proteins into the culture medium and is unable to intoxicate host cells (Fig. 2A and [24], [29]). Expression of wild-type PcrV in PA103Δ*pcrV* restores the regulation of type III secretion and cytotoxicity (Fig. 2A). We screened PA103Δ*pcrV* strains expressing PcrV::EZ in an acute cytotoxicity assay that measures the ability of *P. aeruginosa* to inject a cytotoxin, ExoU. During tissue culture infections, the translocation of this phospholipase toxin results in cell death and loss of the monolayer. The cytotoxic phenotype of derivative strains was screened by a crystal violet retention assay using HeLa-cell monolayers (data not shown). This initial screening step identified 6 nontoxic mutants out of the total of 28 derivatives. We further examined the kinetic patterns of intoxication of HeLa cells using a defined multiplicity of infection (MOI) of 5. The release of lactate dehydrogenase (LDH) within a 3–5 h infection period is a measure of cellular permeability due to the enzymatic activity of ExoU [14]. LDH release was clearly evident when cytotoxic strains were compared with noncytotoxic mutants (compare open/gray to closed symbols in Fig. 2A). There was no significant difference in the overall kinetics of LDH release among a wild-type strain and all of the cytotoxic linker mutants (representative strains are shown in Fig. 2A).

To induce the expression and secretion of type III proteins, *P. aeruginosa* is grown in the presence of a calcium chelator, nitrilotriacetic acid (NTA, [37]). Effector proteins as well as the proteins required for translocation are secreted and accumulate in the extracellular growth medium under the low-calcium conditions [7]. In the absence of a chelator, secretion of effectors is controlled via transcriptional inhibition of the loci involved in the process [7], [16], [38]. The *pcrV*-null strain, PA103Δ*pcrV*, releases type III effectors even in the absence of induction, suggesting that the secretion apparatus is deregulated and constitutively releasing those proteins [29], [38]. Calcium-mediated regulation of T3SS is restored by complementation with *pcrV in trans* [38]. All of our EZ-linker mutants secreted similar amounts of ExoU after growth in the presence of NTA (data not shown). An insertion that blocks secretion of ExoU was not isolated. Nine mutants were calcium insensitive and constitutively secreted ExoU even in the absence of the inducer (Fig. 2B). The expression of these derivatives within bacterial cells was confirmed (Fig. S1). The deregulated-secretion phenotype was defined by an enhanced level (>40%) of ExoU release as compared to the PcrV-complemented strain (Fig. 2B). The remaining derivatives demonstrated a cytotoxic phenotype with a pattern of regulated secretion similar to the complemented strain, which are classified as class I mutants (Fig. 2B and Table 3). Insertion sites in this class of the derivatives were generally located in the N- and C-terminal globular domains with exceptions of EZ154 and EZ155, in which the linker was located at the top region of α-6, one of the coiled-coil helices (Fig. 1 and Table 3).

**Table 3:** Phenotypic classes of PcrV derivatives expressed in the *pcrV*-null strain.

class	strains	cytotoxicity	effector secretion	Characteristics
I	EZ16, EZ28, EZ77, EZ96, EZ101, EZ104, EZ105, EZ107, EZ109, EZ113, EZ116, EZ154, EZ155, EZ179, EZ180, EZ198, EZ202, EZ204, EZ214	Cytotoxic	Regulated	wild-type phenotype
II	EZ119, EZ120, EZ131, EZ134, EZ138, EZ279	Noncytotoxic	Deregulated	*pcrV*-null phenotype
III	EZ44, EZ52, EZ64	Cytotoxic	Deregulated	also deregulated for PopB/PopD secretion
IV	L63A, D133A	Delayed cytotoxicity	Regulated	low secretion of PcrV/PopB/PopD

Insertions associated with the disruption of secretory regulation mapped to two distinct areas of PcrV (Fig. 2B). One set (EZ119, EZ120, EZ131, EZ134, EZ138, and EZ279) mapped to the end of the globular domain and to the central coiled-coil domain (Fig. 1B). The second set (EZ44, EZ52, and EZ64) mapped to the predicted N-terminal globular domain. Interestingly, the derivatives with the constitutive-secretion phenotype segregated into noncytotoxic and cytotoxic groups. Insertions adjacent to or within the coiled-coil region (1^st^ group) prevented both translocation and regulated secretion of ExoU (gray boxes in Fig. 2B). In contrast, insertions in the N-terminal globular domain (2^nd^ group) retained the cytotoxic phenotype (dotted boxes in Fig. 2B). We classified the phenotype of the 1^st^ group (nontoxic) and 2^nd^ group (toxic) of the constitutive-secretion insertions as class II and class III, respectively (Fig. 2B and Table 3).

### Analyses of the protective epitope in PcrV::EZ with translocation-competent cytotoxic phenotype (classes I and III)

In addition to having a role in type III translocation, PcrV is a clinically important protective antigen. Previously more than 70 monoclonal antibodies were screened for protection against ExoU-mediated cytotoxicity and only Mab166 was identified as being protective both *in vitro* and *in vivo* [30]. The protective epitope in PcrV was identified as conformational and the position of the epitope was mapped to a region between amino acids 144 and 257 using truncated molecules or between 158 and 217 as assessed by phage display [30]. Mab166 is reactive to the conformational epitope located in the second globular region between the central and C-terminal helices, α6 and α11, respectively (Fig. 1). The location of the protective epitope in the PcrV-structure models overlaps to the regions identified as the linear and conformational protective epitopes of LcrV [39], [40].

**Figure 3:**
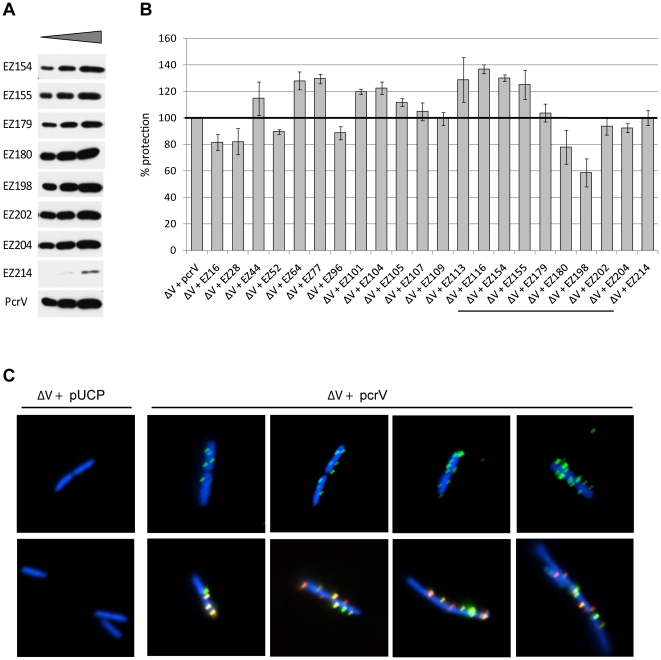
Characterization of translocation-competent derivative proteins and the expressing strains using a protective monoclonal antibody, Mab166. (A) Secretion of PcrV and PcrV::EZ derivatives that contain a linker within a predicted epitope region. V-proteins released into the bacterial culture supernatant under the induced growth condition (+NTA) were detected with Mab166 as a probe. Concentrated supernatant was titrated (2-fold serial dilution) for immunoblot analysis. (B) Retention of the protective epitope analyzed by using a hemolysis-based protection assay. Sheep erythrocytes were infected with Δ*pcrV* mutants in the presence of 10 µg Mab166. The conformational-epitope regions of Mab166 are underlined. (C) Localization of PcrV on the bacterial cell surface detected by immunofluorescence microscopy. PcrV was probed either with rabbit polyclonal IgG alone (shown in green, top panels) or with polyclonal IgG and Mab166 (shown in red and green, respectively; lower panels). Bacterial cells were stained with DAPI (shown in blue).

To determine if this protective epitope was compromised by EZ-linker insertion, monomer forms of all of the PcrV::EZ molecules were initially examined for Mab166 recognition. Western blot analyses demonstrated similar binding of Mab166 to all derivatives, except PcrV::EZ214 (Fig. 3A). EZ214, in which the linker is located between α-9 and β-4 within the epitope region, demonstrated a lower affinity for Mab166. Polyclonal antibody recognizing PcrV bound to all of the EZ derivatives similarly, including EZ214 (data not shown). In order to analyze the retention of the protective capacity of Mab166, 22 translocation-competent mutants were subjected to a hemolysis-based protection assay. Translocator-mediated lysis of erythrocytes is inhibited by Mab166 (data not shown and [27]). In the presence of 10 µg Mab166, hemolysis of erythrocytes infected with ΔV+*pcrV* was reduced approximately 70% (data not shown), which was set as 100% protection. Overall, red blood cells infected with translocation-competent derivatives were protected at an efficiency of 80% or higher compared to the strain expressing wild-type PcrV, ΔV+*pcrV* (Fig. 3B). For EZ214, which possessed a low affinity to Mab166 as detected by Western blot analysis, full protection was observed. For several linker-insertion derivatives, EZ16, EZ28, EZ52, EZ96, and EZ180, there was slight reduction (20% or less) in protection. Protection was compromised only for EZ198 (by 40%, Fig. 3B). This insertion is positioned in the loop structure next to α-8, which is localized to the middle of the mapped epitope region (Fig. 1A and B). These data support the importance of these flexible-loop regions of PcrV for protection from intoxication.

Mab166 has been shown to bind to surface-localized PcrV and inhibit the translocon assembly in host membranes, resulting in a failure to deliver effectors for intoxication [27], [30], [41]. The binding of Mab166 to surface-localized PcrV was analyzed by fluorescence microscopy. First, PcrV was expressed in the ΔV+*pcrV* strain and probed with polyclonal IgG. Spike-shaped signals (shown in green) were detectable on bacterial cell surface (upper panels in Fig. 3C). Bacterial cells were visualized by staining DNA molecules with DAPI (shown in blue, Fig. 3C). Labeling PcrV with both Mab166 (shown in green) and polyclonal IgG (shown in red) resulted in the intense signals of either antibody or both (shown in yellow, lower panels in Fig. 3C). For the *pcrV*-null control strain (ΔV+pUCP), only DAPI-stained cells were visible and no PcrV signals were detected (left panels, Fig. 3C).

### Biochemical analyses of nontoxic class II derivatives and co-expression of PcrV::EZ derivatives with PcrV

**Figure 4:**
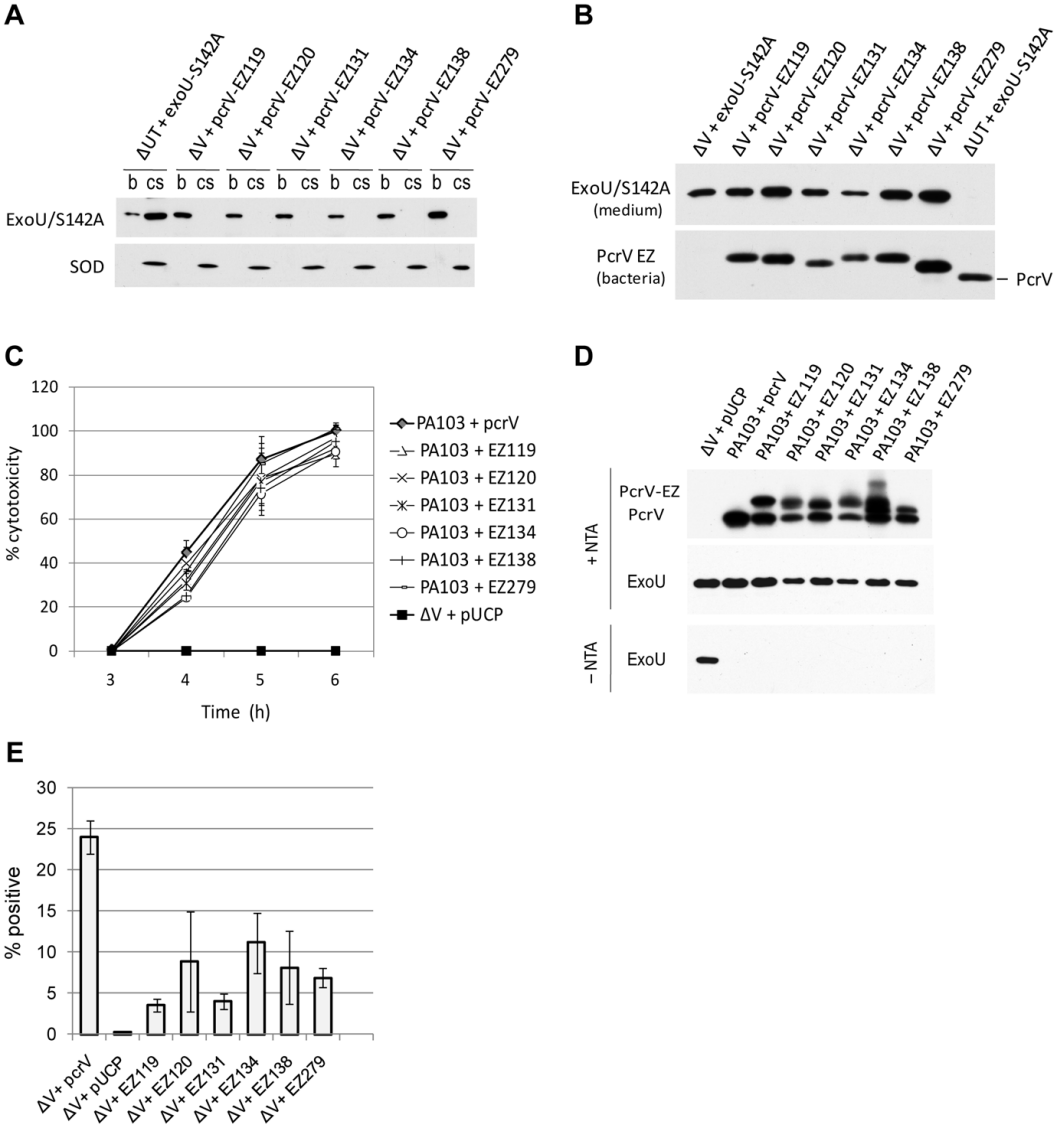
Analyses of class II mutations contributing to the noncytotoxic/constitutive-secretion phenotype. (A) Translocation profiles of ExoU by the EZ-linker mutants during HeLa cell infection. After infection, bacterial cells (b) harvested from the cell culture medium and soluble fractions of HeLa cells (cs) were subjected to Western blot analysis. An anti-SOD antibody was used to detect SOD1 present in HeLa cell soluble fractions. (B) Western blot analysis of ExoU or ExoU-S142A release into cell culture medium during infection. Expression of PcrV::EZ derivatives or parental PcrV in bacterial fractions was detected with anti-PcrV IgG. (C) Cytotoxicity profiles of the strains that co-express the nontoxic derivatives with PcrV. Kinetics of cytotoxicity was measured by LDH release from infected HeLa cells. (D) Immunoblot analysis of secreted PcrV and PcrV derivatives (+NTA) or ExoU (±NTA). Regulation of ExoU secretion was examined when co-expressed with a chromosomal parental copy of *pcrV*. EZ138 contains two cysteine residues in the inserted linker, leading to the extra conformational species observed in the immunoblot (lane 7). (E) Bacterial surface localization of PcrV and class II derivatives. Surface-localized V proteins were quantified by flow cytometry.

The noncytotoxic phenotype observed in six EZ derivatives indicates that these mutants are defective for translocation of ExoU into host cells. To characterize these derivatives further, HeLa cells were infected and then fractionated to analyze the expression and location of the effector protein [bacterial (b), HeLa cytoplasmic (cell soluble, cs), or extracellular medium]. Superoxide dismutase 1 (SOD1) serves as a marker for the HeLa cellular fractions. Strain PA103Δ*exoUexoT*::Tc (PA103ΔUT) expresses none of the known effectors but possesses an intact T3SS (Table 1). After infection with PA103ΔUT expressing a non-catalytic effector protein, ExoU-S142A, the non-catalytic molecule was localized to bacterial fractions and mostly to HeLa cell cytoplasmic fractions (Fig. 4A), confirming that PA103ΔUT is competent for translocation. After infection with the nontoxic PcrV::EZ derivatives, ExoU was undetectable in soluble and insoluble fractions of HeLa cells, supporting the translocation-incompetent phenotype (Fig. 4A and data not shown). The bacterial cells were clearly synthesizing ExoU and PcrV-EZ (Fig. 4A and B). During the infection, ExoU-S142A and ExoU were released to the cell-culture medium in the absence of PcrV (ΔV) and when complemented with EZ-derivatives, respectively (Fig. 4B). No extracellular ExoU-S142A was detected in cellular supernatants from a translocation competent (ΔUT) strain (Fig. 4B, lane 8), suggesting that the effector-translocation machinery is tightly controlled and the bridge between the needle and translocon structures is sealed by PcrV.

The current structural models of the needle-tip complex indicate that multiple units of the V protein oligomerize on top of the needle proteins [34], [42]–[44]. Using a *Yersinia* co-infection system, Marenne *et al.* demonstrated that YopB, YopD, and LcrV are sufficient for channel formation only when these proteins are expressed and secreted by the same bacterium [45]. To determine if the translocation-incompetent PcrV derivatives could exert a dominant effect over wild-type PcrV, six of these proteins were expressed in the wild-type strain, PA103. The ability to deliver ExoU by these co-expression strains was identical to the strain PA103+*pcrV* in LDH kinetic assays (Fig. 4C). Under inducing conditions, all of these strains demonstrated a wild-type secretion pattern of ExoU, PcrV, and PcrV::EZ (+NTA in Fig. 4D). In the absence of NTA-mediated induction, effector secretion was tightly regulated by all coexpression strains similar to the PcrV-complemented strain (–NTA in Fig. 4D). These data suggest that despite expression and secretion from the same bacterium, the function of PcrV was not impaired by translocation-incompetent PcrV::EZ derivatives.

Erythrocytes are often used to identify the formation of T3S translocon channels based on the release of hemoglobins by osmotic lysis of cell membranes [27], [46]. To determine if the noncytotoxic phenotype is mediated by an inability of PcrV::EZ derivatives to form a translocon, erythrocytes were infected with the nontoxic mutants and measured for hemoglobin release. Although the type III-competent strains without effector genes (ΔUT+pUCP and ΔUT+*pcrV*) released hemoglobin, the nontoxic mutants were not hemolytic (data not shown).

A failure of the nontoxic PcrV::EZ strains to form translocons and to influence any phenotypic effect of wild-type PcrV upon co-expression (Fig. 4C and D) implies that the linker insertion impaired the association of the derivatives with the needle components. Surface localization of all of the nontoxic EZ derivatives was 50% or lower than that of PcrV, quantified with either microscopy (Table 4) or flow cytometry analysis (Fig. 4E). These data indicate that the linker insertion within or near the coiled-coil region destabilizes the interaction of the V-tip protein with the needle apparatus.

**Table 4:** Surface localization of PcrV and class II nontoxic derivatives quantified by using immunofluorescence microscopy.

Strain	No. of PcrV/derivative positive cells	No. of bacterial cells	% positive
ΔV+pcrV	54	192	28.1
ΔV+pUCP	2	199	1.0
ΔV+EZ119	19	252	7.5
ΔV+EZ120	24	205	11.7
ΔV+EZ131	12	176	6.8
ΔV+EZ134	24	203	11.8
ΔV+EZ138	19	200	9.5
ΔV+EZ279	21	165	12.7

### Analysis of class III PcrV derivatives with the cytotoxic/deregulated-secretion phenotype

**Figure 5:**
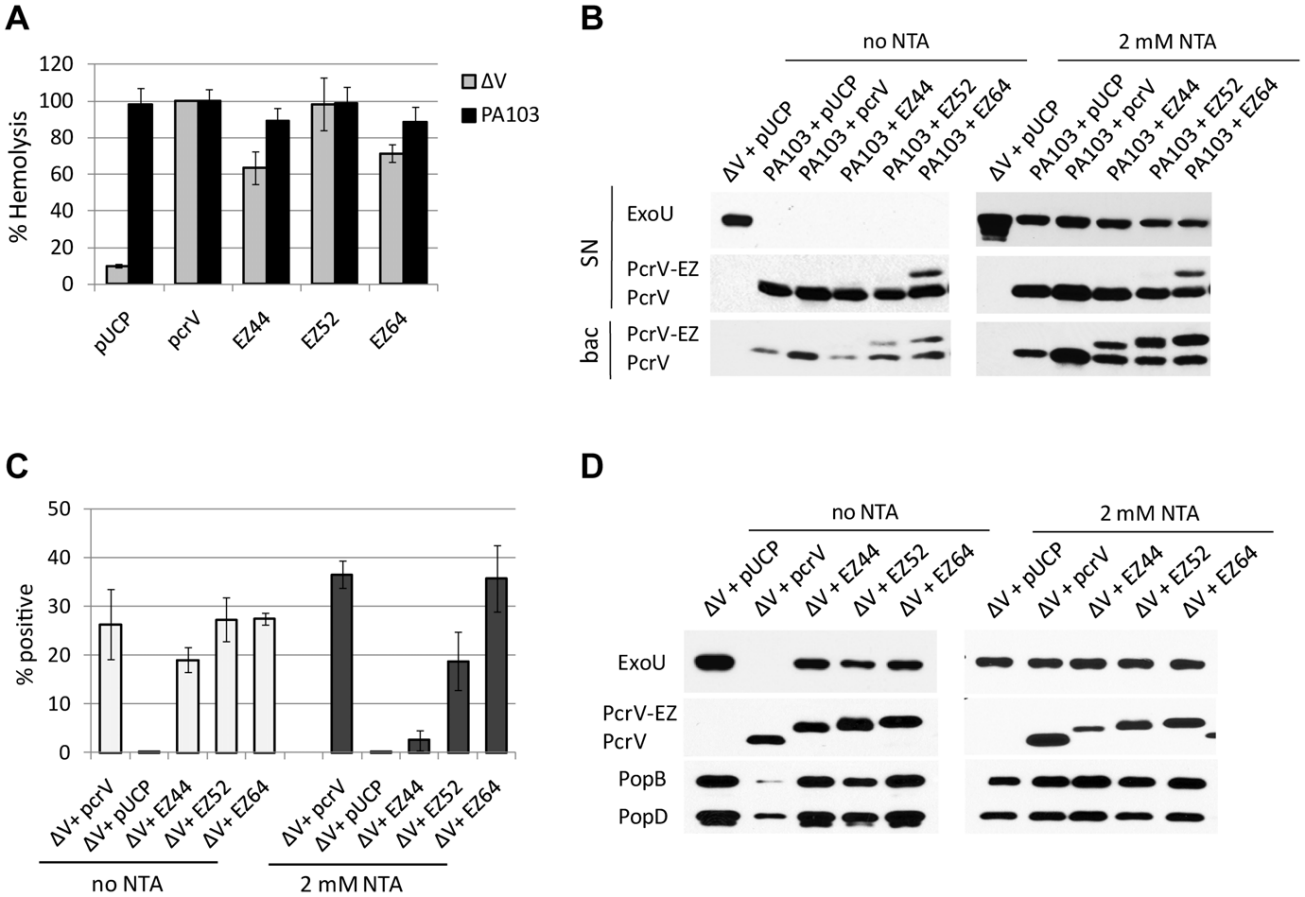
Analysis of class III cytotoxic/deregulated-secretion derivatives possessing a linker insertion in the N-terminal globular domain. (A) Hemolytic activity of EZ44, EZ52, and EZ64 mutants. The derivative proteins were expressed in the either *pcrV*-null (ΔV) or wild-type (PA103) strain. (B) Regulation of ExoU secretion by PcrV::EZ when the proteins were co-expressed in PA103. Under uninduced or NTA-induced conditions, the secretion of ExoU and PcrV/derivatives into growth medium (SN) and the expression of V proteins within bacterial cells were determined by Western blot analysis. (C) Bacterial surface localization of class III derivative proteins. EZ44, EZ52, and EZ64 were expressed in the *pcrV*-null strain (ΔV) in the presence or absence of a secretion inducer, NTA. Surface localization was quantified by flow cytometry. (D) Secretory regulation of ExoU and translocon proteins (PopB/PopD) by class III derivatives, when expressed in the *pcrV*-null strain (ΔV).

The class III strains (EZ44, EZ52, EZ64) demonstrated wild-type levels of cytotoxicity despite possessing the *pcrV*-null phenotype of constitutive secretion (Fig. 2 and Table 3). Their cytotoxic phenotype suggests these derivatives are competent for the assembly of PopB/PopD translocons in host membranes. We used an assay to detect the translocon formation in erythrocytes to determine if these derivative molecules influence the parental PcrV phenotype in a competitive, additive, or synergetic manner. When the proteins were expressed in the ΔV strain, EZ44 and EZ64 demonstrated a 30% to 35% lower amounts of hemoglobin release than the *pcrV* complemented strain (gray bars, Fig. 5A). Expression of these derivatives in the wild-type strain, PA103, demonstrated slightly decreased hemolytic activity compared to the wild-type strains, PA103+pUCP and PA103+*pcrV* (black bars, Fig. 5A). In the case of EZ52, the percentage hemolysis was comparable to the complemented strain (Fig. 5A). Overall, these results indicate that the translocon can still be deployed by EZ44, EZ52, and EZ64 albeit at various efficiencies.

We then analyzed the effect of wild-type PcrV on the constitutive secretion phenotype of class III derivatives. In the absence of NTA, secretion of ExoU by the co-expression strains was as tightly regulated as the wild-type phenotype (SN, Fig 5B). Under inducing conditions (2 mM NTA), levels of ExoU secretion were similar among the co-expression and wild-type strains (SN, Fig. 5B). Amounts of secreted derivative proteins, especially EZ44 and EZ52, were undetectable or extremely low irrespective of NTA (SN, Fig. 5B). When proteins within bacterial cells were probed, co-expression strains possessed similar levels of EZ derivatives and PcrV in the presence of NTA (bac, Fig. 5B). In uninduced conditions, the expression of these proteins within bacteria was low or undetectable (bac, no NTA, Fig. 5B), suggesting the dominant expression and secretion of parental PcrV. Analysis of the molecules in this phenotypic group may help to understand possible differential features of PcrV distinct to translocation or secretion. To further analyze the deregulated secretion/translocation-competent phenotype, class III derivatives were compared for the localization on the bacterial surface by flow cytometry. In the absence of NTA (uninduced), all of the derivatives localized on the bacterial surface similar to PcrV (open bars, Fig. 5C). EZ44 trended towards less surface localization compared to other derivatives but was not statistically significant (*p* = 0.176, *t*-test). Under inducing conditions, the number of bacterial cells with surface-localized EZ64 was similar to the parental control (filled bars, Fig. 5C). Surface localization of EZ44 and EZ52, however, was significantly lower than that of PcrV (filled bars, Fig. 5C). The surface localization of EZ44 was almost as low as the negative control, ΔV+pUCP (Fig. 5C). It should be noted that EZ44 and EZ52 could form translocation channels in erythrocyte membranes and demonstrated a parental pattern of cytotoxicity upon cell contact-mediated type III activation (Fig. 2A and 5A). These results indicate that linker insertions in EZ44 and EZ52 disrupt the association of these molecules with the needle apparatus specifically when low calcium is the activation signal.

Class III derivatives possessing an insertion in the N-terminal globular region lost the control of effector secretion (Fig. 2B). Because PcrV is also required for the secretion of translocon proteins (PopB and PopD), levels of secreted proteins by EZ44, EZ52, and EZ64 were examined by Western blot analysis. Secretion patterns of PcrV and the derivatives into growth medium were similar under either induced or uninduced conditions, except a slightly reduced secretion of EZ44 in the presence of NTA (Fig. 5D). The class III phenotype of deregulated secretion of ExoU was recapitulated for these derivatives (no NTA, Fig. 5D). Levels of secreted PopB and PopD by all strains were indistinguishable in the presence of NTA (Fig. 5D). After growth in the absence of NTA, the complemented strain (ΔV+*pcrV*) secreted PopB and PopD at fairly low levels while the *pcrV*-null strain and class III mutants secreted these translocators at high levels similar to the induced condition (Fig. 5D). These results suggest that the insertion in EZ44, EZ52, and EZ64 disrupts the low-calcium dependent control of the secretion mechanism for both effector and translocon proteins.

### Cytotoxic phenotypes of single site-specific mutations (class IV)

**Figure 6:**
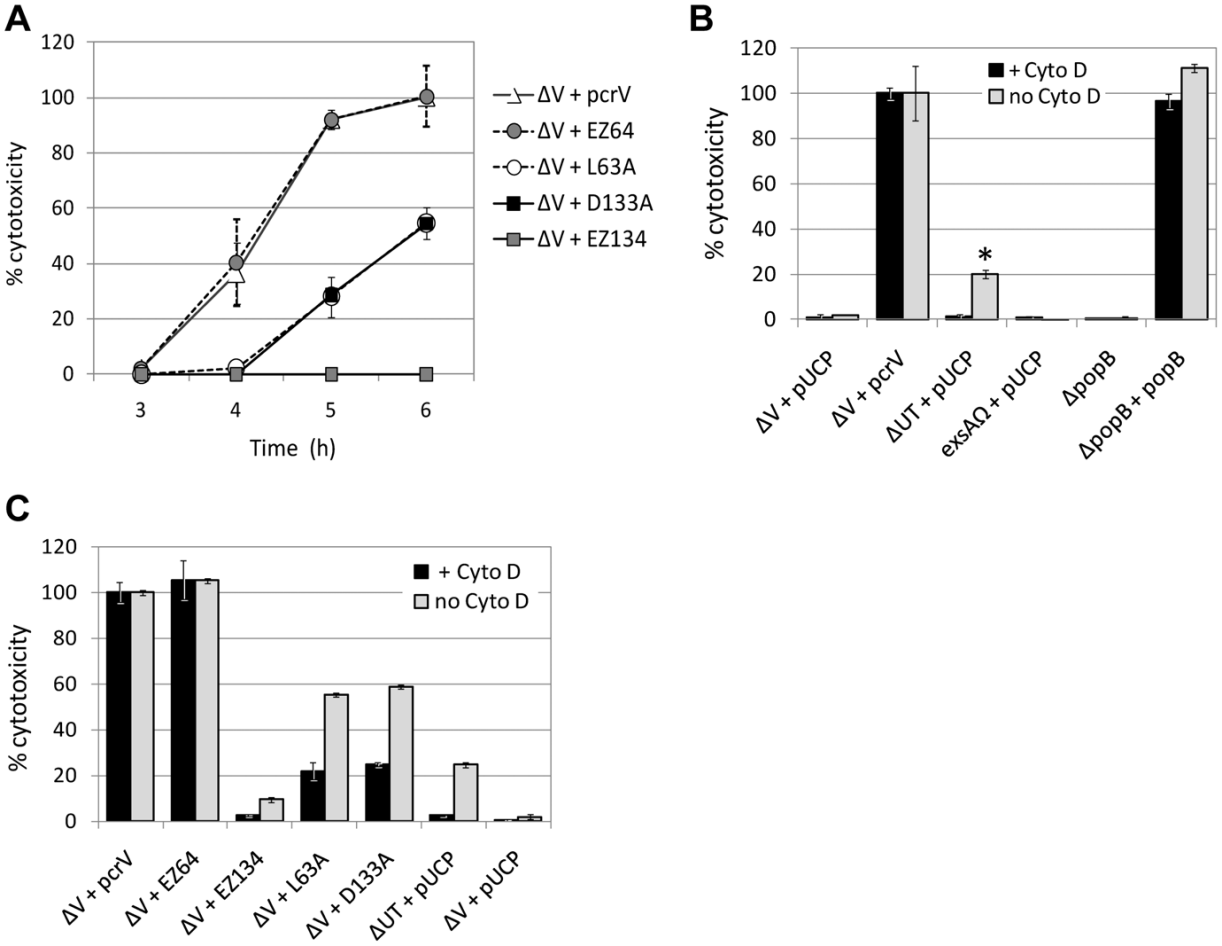
Cytotoxicity profiles of class IV site-specific mutants compared to linker-insertion strains. (A) LDH release comparison of the point mutants (L63A and D133A) and the strains containing an EZ-linker insertion in adjacent sequence locations. (B) Cytotoxic effects on HeLa cells caused by formation of type III translocation channels at 7 h post-infection. LDH release from HeLa cells infected with PA103ΔV+pUCP, ΔV+*pcrV*, ΔUT+pUCP (competent type III injectisomes without the expression of any effectors) and PA103*exsA*Ω+pUCP (type III incompetent). The *popB*–deletion mutant and its complemented strain were used to represent the effect of a translocon protein on cytotoxicity. LDH release was tested in the presence (black bars) or absence (gray bars) of cytochalasin D (3 µg/ml). Cytochalasin D was added to examine the effect on the type III channel-mediated cytotoxicity and ExoU-mediated killing. *p<0.001 by t-test, compared to ΔV+pUCP. (C) The effect of cytochalasin D on the delayed cytotoxicity of class IV mutants. Cytotoxicity was measured and presented as (B).

Two linker sites, representing the constitutive-secretion/cytotoxic phenotype (EZ64) and the constitutive-secretion/noncytotoxic phenotype (EZ134), were selected for site-directed mutagenesis. Alanine was used to replace a leucine residue at position 63 (L63A) and an aspartic acid residue at position 133 (D133A). When LDH release was monitored during an infection, both site-specific mutants appeared to have a delayed-cytotoxic phenotype and classified to the forth phenotypic group, class IV (Fig. 6A and Table 3). The time lag prior to the intoxication by these mutants was approximately 1 h, and after the lag period, the kinetics of LDH release was similar to the complemented strain (Fig. 6A).

Formation of translocation channels in eukaryotic plasma membranes by effectorless strains can be detected by measuring osmotic lysis via the release of LDH from infected cells [45], [47]. To monitor translocon formation in the HeLa cell membrane, the release of LDH was measured at 7 h post-infection with various strains of *P. aeruginosa*. HeLa cell infection with a T3SS competent and effectorless strain, PA103ΔUT+pUCP, demonstrated a statistically significant level of LDH release (*p*<0.001 by t-test, Fig. 6B). Strain PA103*exsA*Ω is unable to express the T3SS due to a mutation introduced into a key transcriptional activator, ExsA (Table 1) [7], [48]. Release of LDH was undetectable after infection with this strain (Fig. 6B). Collectively, these data support the hypothesis that lysis of HeLa cells in the absence of effectors requires the intact T3SS.

To determine whether the cytotoxic phenotype was specifically related to an intact translocation operon, translocator-deletion mutants PA103Δ*pcrV* and PA103Δ*popB* were compared to PA103ΔUT in the presence or absence of an inhibitor of actin polymerization, cytochalasin D (Fig. 6B). Viboud and Bliska demonstrated that treatment with cytochalasin D reduced type III-mediated osmotic lysis of eukaryotic cells during infection with *Yersinia* [47]. Protection by this inhibitor is mediated by the prevention of local actin rearrangements at the insertion sites of YopB/YopD translocons, where cytochalasin D is postulated to suppress disruptive membrane movement [47]. When cells were infected with translocator-deletion mutants, Δ*pcrV* and Δ*popB*, LDH release was at a basal level similar to the T3S incompetent strain, PA103*exsA*Ω (Fig. 6B). When cytochalasin D-treated cells were infected with an effectorless strain of *P. aeruginosa*, LDH release was ameliorated (ΔUT+pUCP in Fig. 6B), indicating that the observed LDH release is due to membrane disruption caused by insertion of PopB/PopD translocons. Complementation of translocator-deletion mutants, ΔV+*pcrV* and Δ*popB*+*popB*, allowed intoxication with ExoU even in the presence of cytochalasin D (Fig. 6B). The inhibitor treatment did not interfere with the translocation of effectors (Fig. 6B and [49],[50], [51], suggesting that cytochalasin D does not inhibit insertion of translocons into the eukaryotic membrane. These results confirm that both PopB and PcrV translocators are required for the channel formation followed by translocation of the effector.

The delayed-toxic phenotype of class IV mutants could be mediated by the inefficient formation of translocons in the host plasma membrane. At 7 h post infection of HeLa cells, the point mutants released intermediate levels (∼60%) of LDH, not as high as the fully toxic strains but not as low as the effectorless type III-competent strain, ΔUT+pUCP (gray bars, Fig. 6C). The intermediate levels of LDH release by both point mutants were partially inhibited by the addition of cytochalasin D (black bars, Fig. 6C). This inhibitor could not reduce ExoU-mediated LDH release even at early stages of cellular intoxication (data not shown). These data suggest that class IV mutants are capable of forming translocons but the slow kinetics of the channel formation allows cytochalasin D to be effective prior to ExoU translocation.

### Biochemical analyses of class IV mutations for translocon formation and secretory regulation

**Figure 7:**
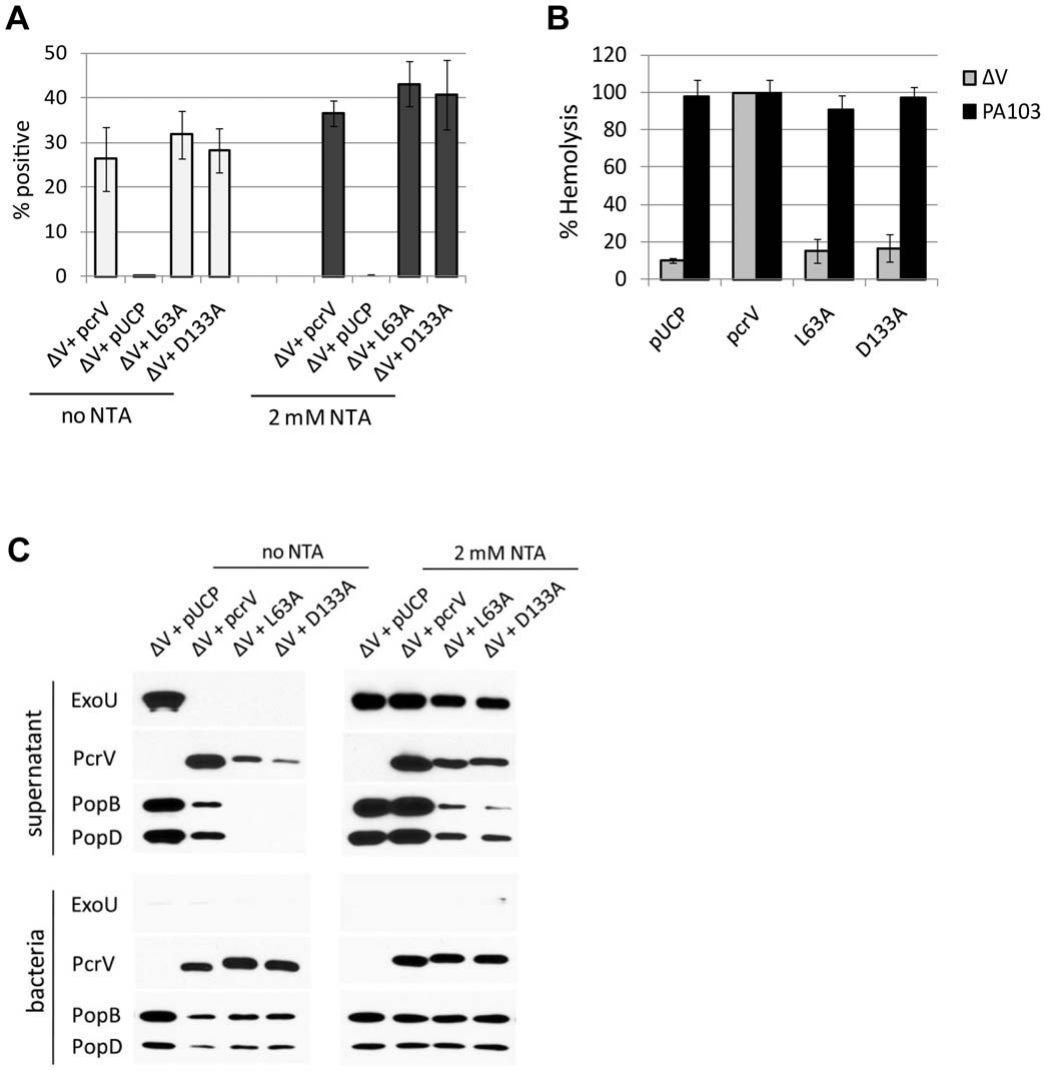
Biochemical analyses of class IV derivatives containing a point mutation. (A) Bacterial surface localization of the derivatives containing an alanine substitution quantified by flow cytometry. L63A and D133A proteins were expressed in the *pcrV*-null strain (ΔV) in the presence or absence of NTA. (B) Measurement of hemolytic activity of the *pcrV*-point mutants. Sheep erythrocytes were infected for 1 h with either PA103 or the ΔV strain expressing the L63A or D133A derivative. (C) Regulation of effector and translocator secretion by class IV derivatives expressed in the *pcrV*-null strain (ΔV). Under the uninduced (no NTA) or induced (2 mM NTA) condition, proteins secreted into growth medium (supernatant) were detected by Western blot analysis. Expression of the proteins within bacterial cells was shown as comparison (bacteria).

HeLa cell infection with ΔV+L63A and ΔV+D133A strains demonstrated the delayed-cytotoxicity phenotype (Fig. 6A and C). The release of LDH by these strains was partially inhibited by cytochalasin D (Fig. 6C), suggesting that the low expression or slower deployment of translocon proteins (PopB and PopD) due to the alanine substitution. To determine if L63A and D133A proteins are adequately expressed and associated with the type III needle, bacterial surface localization of the derivatives was analyzed by flow cytometry. The number of bacterial cells with surface localized L63A and D133A was comparable to the parental control, irrespective of the presence of NTA (Fig. 7A). These results implicate that the delayed-cytotoxic phenotype is related to a malfunction of these derivatives other than insufficient protein expression or surface localization.

To evaluate the formation of translocon channels in membranes, sheep erythrocytes were infected with a point-mutation strain ΔV+L63A or ΔV+D133A. Infection with either of the strains released only basal levels of hemoglobin, similar to the *pcrV*-null strain at a 1 h incubation time (gray bars, Fig. 7B). The hemolysis data along with the delayed cytotoxic effect on HeLa cells (Fig. 6A and C) suggest a slower rate of the insertion and assembly of translocons in host membranes by these mutants. Co-expression of the derivatives with PcrV in the parental PA103 strain restored hemolytic activity to the levels of positive control strains (filled bars, Fig. 7B). These results indicate that L63A and D133A derivatives do not possess a dominant negative effect on the translocon assembly by parental PcrV.

Class IV derivatives appeared to require additional time for the assembly of translocation channels, which can be mediated by low expression or secretion of translocator proteins. For the point mutants, PcrV, PopB, and PopD were similarly expressed and retained within bacterial cells at the level of the ΔV+*pcrV* strain under induced and uninduced conditions (bacteria, Fig. 7C). In contrast, levels of secreted PcrV, PopB, and PopD by these mutants were significantly reduced (supernatant, Fig. 7C). Notably, secreted PopB and PopD proteins were undetectable in the absence of NTA (no NTA, Fig. 7). In a calcium-rich environment, expression and secretion of ExoU were tightly controlled by PcrV, L63A, and D133A and the regulatory system was perturbed only when *pcrV* was deleted (no NTA, Fig. 7C). Under low-calcium inducing conditions, most of expressed ExoU was secreted into the growth medium and barely detectable in bacterial cell fractions of all four strains (2 mM NTA, Fig. 7C). These data suggest that the alanine substitution at L63 or D133 decreases the efficiency of translocator secretion, resulting in the slow translocon assembly and delayed-cytotoxic phenotype.

## Discussion

The T3SS is a key virulence determinant of *P. aeruginosa* and multiple other Gram-negative organisms. Understanding the essential components and functional mechanisms of this intoxication system will be important for the development of novel therapeutics that could inhibit translocon assembly, toxin translocation, and signaling activity associated with transcriptional induction in response to contact with eukaryotic cells [33], [52]–[54]. This study focuses on a linker-scanning mutagenesis analysis of PcrV, the needle-tip protein of the *P. aeruginosa* type III apparatus. Despite the molecular, biochemical, and microscopic analyses of the tip complexes in the past, the multifunctional nature of tip proteins makes it difficult to tease out how they mechanistically serve as regulators for expression and secretion of effectors as well as extracellular chaperones for integration of the hydrophobic translocators (PopB and PopD) into eukaryotic plasma membranes. Understanding the functional mechanism of the needle-tip proteins is particularly relevant to the design of vaccines and therapeutics targeted to neutralize activities that contribute to intoxication of host cells [29], [33], [55]–[57].

**Figure 8:**
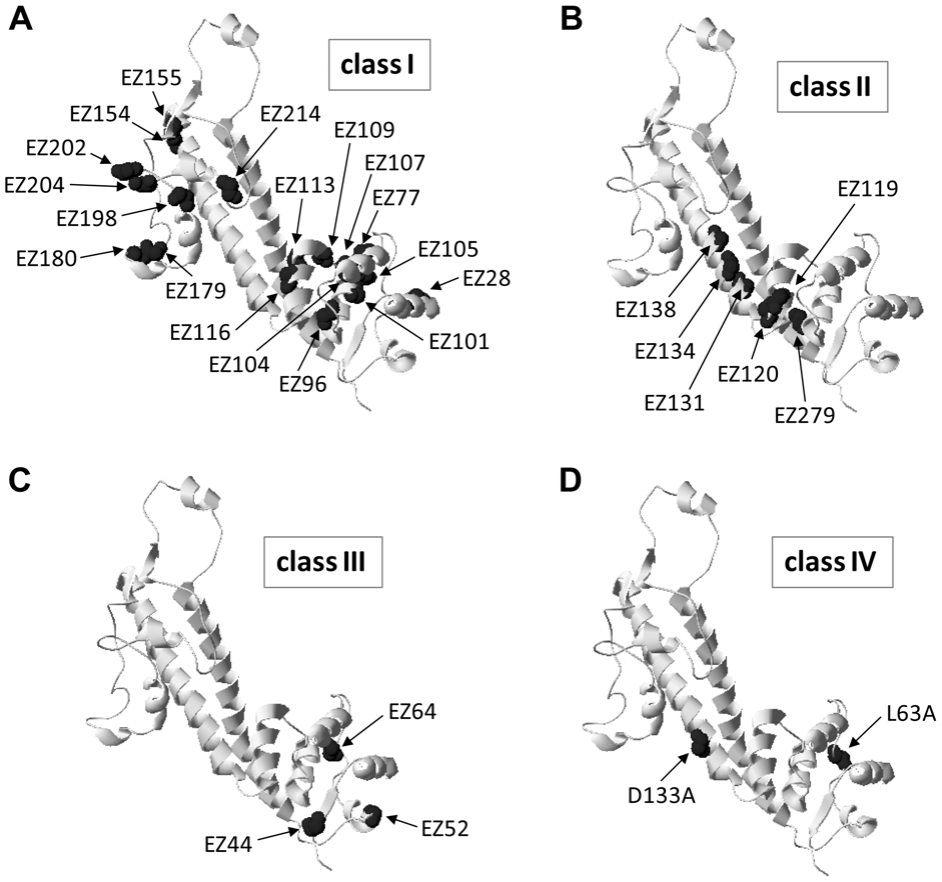
PcrV tertiary structure models indicating the insertion/mutation sites responsible for the phenotypic class. (A) Class I insertions: the wild-type cytotoxic/regulated-secretion phenotype. The residue, at which an EZ-linker is inserted, is shown in black. (B) Class II insertions: the *pcrV*-null noncytotoxic/deregulated-secretion phenotype. (C) Class III insertions: cytotoxic/deregulated-secretion phenotype. The linker is inserted within the N-terminal globular domain. (D) Class IV mutations: delayed-cytotoxicity/regulated-secretion phenotype. Although secretion of an effector protein is well controlled, the secretion levels of translocators (PcrV derivative, PopB, and PopD) are decreased by a single amino-acid substitution.

The linker-scan analysis of PcrV was initiated as an unbiased approach to cause distinct 19 amino-acid insertions in this molecule. The lower frequency of C-terminal insertions does not correlate with the selection process relative to protein expression as the clones were screened in *E. coli*, a host in which the *Pseudomonas*-specific pS promoter does not initiate transcription [58]. Most of the insertions (∼79%) did not disrupt the function of effector translocation, retaining the cytotoxic phenotype of the strains. Amino acid sequences within the insertion grouped into 3 patterns flanked by various sequences due to the target site duplication (Table 2). Each group of linkers contains either 3 cysteine residues, 2 cysteine residues, or a proline residue, which may introduce a circular or folded structure of an EZ linker by a formation of disulfide bonds or a proline kink in the middle of the linker. PcrV possesses no cysteine residues to form a covalent bond with a free cysteine in the linker. Biochemical and biological analyses of the linker-insertion derivatives identified four types of phenotypic groups possessing distinct structural and functional regions of PcrV (Table 3 and Fig. 8).

### Class I insertions (wild-type phenotype; Figure 8A)

Most of the insertions for this phenotypic group are mainly positioned in N- and C- terminal globular domains, except EZ154 and EZ155 (Table 3 and Fig. 8A). These two insertions are located at the tip end of the central coiled-coil structure, less likely involved in the coiled-coil interaction. Collectively, both N- and C-terminal globular domains are more permissive to insertions, indicating the structural flexibility in those domains.

PcrV is a clinically important molecule as an effective protective antigen for *P. aeruginosa* infection. All of the PcrV::EZ proteins were immunoreactive to Mab166 examined by Western blot analysis, except EZ214. Using an erythrocyte-protection assay, we demonstrated that most of the translocation-competent mutants retain the conformational protective epitope. Mab166 protected against infection with the strain expressing EZ214, suggesting the epitope in this molecule is preserved during bacterial contact with erythrocytes. Reduced protection for EZ198 is understandable since the linker-insertion site is located within the predicted epitope region. These protection and biochemical data indicate that the loop regions proximal to short helices (α8 and α9) are critical for the recognition and protection by Mab166 and that the epitope region appears to be structurally flexible and retains the protective role irrespective of the 19-residue insertions.

### Class II insertions (pcrV-null phenotype; Figure 8B)

The *pcrV*-knockout deletion in the chromosome of PA103 loses the regulatory function for both secretion and translocation of effectors, resulting in a failure to intoxicate host cells and constitutive secretion of effector proteins into a growth medium. The regulatory mechanism of T3SS is complex. It has been proposed that there are multiple-levels of control by the needle-tip complex, needle proteins, and cytosolic regulators located around the base of a type III apparatus [28], [42], [45], [59]–[61]. For example, cytoplasmic chaperones for PcrV and LcrV (PcrG and LcrG, respectively) are involved in secretory regulation of *P. aeruginosa* and *Yersinia* species [59], [60]. In the case of *P. aeruginosa* strain PAO1, a deletion of either *pcrV* or *pcrG* leads to a calcium-blind secretion phenotype, and the deregulation effect is additive for the double-deletion mutant [60]. In *Yersinia* species, YopN with its adaptor TyeA also acts as a cytoplasmic regulator in addition to LcrG [28], [45]. Moreover, it is postulated that needle proteins are involved in conducting an activation signal bidirectionally to coordinate the regulation of T3SS [42], [61]. Further studies are needed to elucidate how the secretion and translocation mechanisms are intricately accommodated by various regulatory proteins present in distinct locations.

The class II deregulated-secretion/translocation phenotype observed when *pcrV* is deleted from strains PA103 and PAO1 is recapitulated in *Shigella* species when the V-like gene, *ipaD*, is deleted [60], [62], [63]. In contrast, a knockout or partial deletion of *pcrV* in *P. aeruginosa* CHA and PAK strains or the *lcrV* gene in *Yersinia* species retains the regulated-secretion phenotype despite the loss of cytotoxicity [25], [64]–[66]. This phenotypic diversity may be specific to the strain [24] or the size and region of the deletion if the entire gene is not deleted.

Our data demonstrated that the inability to deliver effectors by class II mutants is due to a failure of the PcrV derivatives to localize on the bacterial surface. The results suggest that class II derivatives form a structurally unstable complex possessing a low affinity to the needle apparatus or they are unable to oligomerize to form a needle-tip complex. We expressed class II derivatives in a wild-type host background with the expectation of hetero-oligomer formation with PcrV. The attenuated affinity of the derivative proteins for the needle structure, however, allowed wild-type PcrV to be functionally dominant, selecting for parental phenotypes during co-expression.

An insertion region that contributes to the class II phenotype is mapped to the end of N-terminal globular domain (EZ119, EZ120) and at the base of the intramolecular antiparallel coiled-coil structure (EZ131, EZ134, EZ138, EZ279) shown in Fig. 8B [34]. Many residues conserved in both PcrV and LcrV are located on the helices that form the coiled-coil structure, suggesting a structural importance in these regions. Caroline *et al.* showed that one of their site-specific mutations (L276A) in one of the coiled-coil helices of PcrV reduced the toxic effect of this strain on macrophages *in vitro* [23]. Lee *et al.* demonstrated that single amino acid replacement of the F279 residue of PcrV to arginine abolishes the cytotoxic phenotype [60]. Combined with our data, hydrophobic residues in this region of PcrV must be critical for stabilizing the structure. Interestingly, the intramolecular coiled-coil motif is also seen in structural analyses of the needle subunit proteins, suggesting a common mechanism of assembly of tip proteins and needle subunits [28]. The C-terminal α-helix of LcrV, one of the coiled-coil helices, is structurally homologous to a stabilizing helix of the *Shigella* needle protein (MxiH), which permitted the modeling of the LcrV-pentamer tip complex on the top end of the MxiH needle without structural restraints [42]. Thus, we suggest that the disruption of the coiled-coil structure by a linker insertion leads to a failure to assemble or to position the V-tip complex on the type III needle, resulting in the class II *pcrV*-null phenotype.

### Class III insertions (cytotoxic/deregulated-secretion phenotype; Figure 8C)

Insertions between amino acids 44 and 64 of PcrV imparted the class III phenotype of cytotoxicity with constitutive secretion (Table 3 and Fig. 8C). Insertions in this area compromised the secretory regulation of the effector, ExoU, as well as translocon proteins, PopB and PopD. Despite of the defect in secretory control, the derivatives (EZ44, EZ52 and EZ64) retained the capability to assemble translocons and translocate the effector, resulting in wild-type levels of cytotoxicity. This secretory-control region is located within an N-terminal globular domain, which is involved in associating with the needle proteins as it is part of the base of the V-tip complex [34]. Our flow cytometry analysis demonstrated that EZ44 and EZ52 molecules are localized on the bacterial surface in the uninduced environment but delocalized in low-calcium induced conditions. These data suggest that this region is involved in the mechanical control of the type III apparatus responding to environmental signals. Intriguingly, the insertion is tolerated for translocation events, suggesting that different conformations of PcrV exist for secretion and translocation functions. We postulate that the conformations of this needle-tip protein are distinct in response to the activation stimuli of the type III machinery specific to contact-dependent translocation (a well-coordinated process with tightly sealed transport of effectors) or chelator-induced secretion (less-controlled release of translocators and effectors).

Structural changes within this N-terminal region may influence the regulation of the opening/closing of the tip complex in addition to the affinity of the tip protein for the injection needle [42], [65], [67]–[69]. Furthermore, the conformation of the V proteins for translocation processes may be distinct to each stepwise function, such as insertion of translocators into membranes, assembly of a translocon, and delivery of effectors. Our data support a model in which PcrV secretes translocators at a basal level but not an effector until receiving a signal based on the host contact or low calcium environment [28], [49]. Elper *et al.* and others demonstrated that IpaD, a tip protein in *Shigella*, senses small molecules (deoxycholate or other bile salts) that stabilize the translocator complex prior to T3SS induction [70], [71].

The deregulated-secretion phenotype of class III derivatives is overpowered by functional PcrV when proteins are co-expressed in wild-type PA103, resulting in tightly regulated secretion of ExoU. It is notable that the amount of secreted derivative proteins is significantly lower than that of PcrV even though the expression levels of all proteins are equivalent. When the derivatives were expressed in the *pcrV*-null host strain, secretion of these proteins was not inhibited. These data imply that wild-type PcrV is a preferred substrate for the secretion machinery and regulators involved in protein export.

The tip complex formed by the derivatives, EZ44, EZ52 and EZ64, is competent to control the insertion of the hydrophobic translocators in host plasma membranes and the assembly of the translocon structure. For the needle-tip proteins from *Shigella* (IpaD) and *Burkolderia* (BipD), the N-terminal domain is believed to function as an intramolecular chaperone to control the timing of self-oligomerization [72]. However, the structure of the N-terminal domain of PcrV and LcrV is bulky and has few structural similarities to IpaD and BipD [72]. In addition, no chaperones specific to IpaD and BipD have been found, while PcrV and LcrV have the cognate chaperones PcrG and LcrG that are expressed from the same operon. It is postulated that PcrV and LcrV act as chaperones for translocon proteins [27]. Specific domains of PcrV may perform chaperone functions to control the self-oligomerization of PcrV and assemble the PopB/PopD translocon in the eukaryotic membrane. More studies are needed to test these hypotheses.

### Class IV mutations (delayed-cytotoxicity/regulated-secretion phenotype; Figure 8D)

Site-specific alanine substitutions (L63A and D133A) made in two regions of PcrV demonstrated a class IV delayed-cytotoxic phenotype (Table 3 and Fig. 8D). In this case, we believe that translocon proteins are deployed and that translocation of ExoU occurs as cytotoxicity is observed even in the presence of cytochalasin D. At the late time point of HeLa cell infection, levels of LDH release by these mutants are higher than by an effectorless type III-competent strain, indicating that cytotoxicity is mediated by the formation of translocation channels and the enzymatic activity of translocated ExoU. It is unclear why a site-specific lesion (L63A) delayed in the induction of cytotoxicity relative to the linker counterpart (EZ64), which has a cytotoxic phenotype. The leucine at residue 63 is conserved and positions next to an LxxLxxL zipper motif, which is implicated for the interaction with other molecules, possibly a needle protein. The other residue, D133, is located within another leucine motif (Lx3Lx2Lx3L) important for the formation of the intramolecular coiled-coil structure [73].

Co-expression analysis of class IV derivatives with PcrV demonstrated the retention of the parental phenotype. L63A and D133A molecules may not oligomerize with themselves or with PcrV due to a loss of a hydrophobic- or charged-side group in the residue by alanine substitution. Amounts of the derivatives secreted by these mutants were extremely low, which may explain the dominant effect of wild-type PcrV on translocon assembly at the initial stage of erythrocyte infection. The alanine replacement may also influence the association of PcrV with its cognate chaperone, PcrG, in the bacterial cytoplasm. It is postulated that the interaction between PcrV and PcrG facilitates the export of PcrV, yet the interaction is not required for the secretory regulation of effectors [60]. If the affinity to PcrG is decreased, the PcrV derivatives may be inefficiently unfolded prior to the export process, leading to the delayed establishment of translocon structure and effector translocation.

Our biochemical analyses indicated that the expression patterns of all of the translocators and the surface localization of the V derivatives are similar to those of PcrV. However, secretion levels of translocators, especially translocon proteins PopB and PopD, were extremely low regardless of the presence or absence of NTA. Erythrocytes infected with these point mutants did not release hemoglobin during the 1 h-incubation time, indicating the slower kinetics of PopB/PopD deployment. Once translocons were formed, the kinetics of ExoU-mediated cytotoxicity was equivalent to the parental strain. It is intriguing that the delayed-secretion phenotype specific to translocator proteins was also observed with an *mxiC* mutant in *Shigella* [61]. MxiC is a cytoplasmic regulator for a *Shigella* type III system, which belongs to the family of YopN/TyeA in *Yersinia*. A knockout mutant of *mxiC* constitutively secretes effectors but the secretion of IpaC, a PopD ortholog translocator, is weak and delayed upon type III induction [61], [74]. These phenotypic properties combined with our data suggest that the control of type III machinery is coordinated specific to the type of secretion substrates, either translocators or effectors. Further studies are needed to elucidate the control mechanisms of the T3SS distinct to translocators and effectors.

### Conclusions

Comprehending how the needle-tip complex interfaces with the type III machinery and the translocon assembles in eukaryotic membranes will be important towards understanding the T3SS-mediated intoxication mechanisms and protective nature of antibodies to V antigens. The tertiary structure models of tip proteins provide a template to design V antigens that retain immunogenicity but lack immunosuppressive properties [55], [75]. We isolated the strains with interesting biological and biochemical phenotypes: constitutive secretion with cytotoxicity, delayed cytotoxicity, delocalization of the tip protein only upon low-calcium induction, and low secretion levels of translocators but not of effectors. Further analyses of PcrV and the derivatives compared to the homologous tip proteins in the Ysc family, LcrV (*Yersinia*) and AcrV (*Aeromonas*), as well as those belonging to other families, IpaD (*Shigella*), BipD (*Burkolderia*), SipD (*Salmonella*), and EspA (enteropathogenic *E. coli*), will advance a mechanistic understanding of how these molecules function in the secretion, translocation, and intoxication processes. Furthermore, it is important to elucidate the control mechanism of the type III system, which is coordinated by V-like proteins, cytoplasmic regulators, and needle proteins. The functional and regulatory information of this multifunctional translocator, PcrV, will facilitate the development of new-generation vaccines and therapeutics targeting on type III-mediated intoxication of host cells.

## Materials and Methods

### Bacterial strains and growth in bacteriological medium

*P. aeruginosa* strains used in this study are shown in Table 1. The TransforMax™ EC100™ *Escherichia coli* strain (*F^−^ mcrA* Δ*(mrr-hsdRMS-mcrBC)* ϕ*80dlacZΔM15 ΔlacX74 recA1 endA1 araD139 Δ(ara, leu)7697 galU galK λ^−^ rpsL nupG*) from EpiCentre was used in this study. *P. aeruginosa* strains were cultured on Vogel-Bonner minimal medium [76] agar plates in the presence or absence of 400 µg/ml carbenicillin at 37°C. *E. coli* strains were cultured on Luria agar plates or broth supplemented with 50 µg/ml kanamycin, and/or 100 µg/ml ampicillin as required.

### Random mutagenesis by linker insertion

Random mutagenesis of *pcrV* was performed using the EZ::TN™ In-Frame Linker Insertion Kit, according to manufacturer's instructions (EPICENTRE). The plasmid pUCP-pS-*pcrV*, an *E. coli-Pseudomonas* shuttle vector carrying *pcrV* under the control of the *exoS* promoter (pS, [29]), was used as template DNA for the *in vitro* transposition reaction. After the transposition, the reaction mixture was electroporated into *E. coli* and grown on Luria agar containing 50 µg/ml kanamycin and 100 µg/ml ampicillin to select for clones carrying transposed plasmids. Plasmid DNA was prepared and subjected to restriction mapping and DNA sequence analysis using primers FP-2 and RP-2 provided in the linker insertion kit to determine the site of insertion of the EZ::TN™ <*Not*I/KAN-3> transposon in the *pcrV* coding region. Plasmids carrying insertions in *pcrV* were subjected to *Not*I digestion and re-ligation to remove all but 57-base pairs of insertion-derived sequence resulting in the in-frame insertion of 19 codons into *pcrV*. Each resolved clone was subjected to DNA sequence analysis to confirm the in-frame insertion. Plasmids carrying the 19-codon insertion in *pcrV* were transformed into *P. aeruginosa* strains, PA103Δ*pcrV* or PA103.

### Site specific mutagenesis

Plasmid, pUCP-pS-*pcrV* was purified using a Qiagen miniprep kit. Plasmid DNA was mutagenized using Change-IT™ kit (USB). Primers used are (underline shows difference from wild-type sequence):

L63A: 5′-TCAAGGCGCTCGCCTGGTTGGCCGCGGCCAATCCGTCCGCGC-3′

D133A: 5′-CAAGCGCAAGGCGCTGCTCGCCGAGCTCAAGGCGCTGACCG-3′

Colony screening PCR and restriction enzyme digestion of the plasmids were used to select point mutants of interest. The mutations were confirmed by DNA sequencing analyses.

### Expression and controlled secretion of type III proteins in bacterial culture

*P. aeruginosa* strains were grown in Luria broth (LB) containing 400 µg/ml carbenicillin in the presence (inducing condition) or absence (non-inducing condition) of 2 mM NTA. Culture supernatant was separated from bacterial cells by centrifugation and subjected to ammonium sulfate precipitation [37]. After precipitation, the concentrated proteins were normalized to an equivalent OD_540_ by suspending in SDS-PAGE sample buffer.

### Protein structure modeling

The amino acid sequence of PcrV from strain PA103 used in this study was submitted to Swiss Model server (Automated Comparative Protein Modeling Server, http://swissmodel.expasy.org). The structural data of the ortholog LcrV from *Yersinia* (PBD: 1r6f chain A) was used as a template for modeling. The Swiss-Pdb Viewer 4.0 was used to indicate the sites of the linker insertion in the PcrV structural model [36]. For coloring, the secondary structure succession mode was used to distinguish alpha-helices and beta-sheets. LcrV sequence from *Y. pestis* strains KIM 10 (NP_857946 and NP_857751) and Z176003 (YP_003560525) was used for alignment.

### Western blotting

For detection of specific proteins, samples were separated by SDS-PAGE, transferred to nitrocellulose membranes, and probed with rabbit anti-PcrV IgG, sheep anti-SOD IgG (CalBiochem), rabbit anti-PopD IgG, or mouse anti-ExoU monoclonal antibody U29F8. PopB was detected with a mouse monoclonal antibody produced by using a transmembrane domain-deleted molecule (a gift from Dr. Joseph T. Barbieri, Medical College of Wisconsin). As a secondary antibody, horseradish-peroxidase conjugates for rabbit IgG (Sigma), sheep IgG (Jackson), or mouse IgG (Roche) were used to detect the appropriate primary antibody. SuperSignal West Pico Chemiluminescent Substrate (Pierce) was used for detection of immuno-reactive proteins. Signals from Western blot chemoluminescent analyses were quantified by using an AlphaImager AIC (Alpha Innotech) for densitometry.

### Lactate dehydrogenase (LDH) release assays to measure cytotoxicity

HeLa cells were grown in 6-well tissue culture plates to approximately 6×10^5^ cells per well. Bacterial cells suspended in serum-free DMEM were added for infection with an MOI of 5. When required, HeLa cells were pre-incubated for 30 min in serum-free DMEM containing 3 µg/ml cytochalasin D prior to and during infection. To follow the release of LDH over time, 30 µl aliquots of the tissue culture supernatant were removed at various time points and subjected to centrifugation. The supernatant was diluted in serum-free DMEM and LDH activity was measured using the CytoTox 96® Non-Radioactive Cytotoxicity Assay (Promega) according to the manufacturer's instructions. Percent cytotoxicity was calculated using PA103Δ*pcrV*+pUCP-*pS-pcrV* as the maximum reference and uninfected samples as the baseline. Assays were performed at least 3 times as independent experiments and the error bars indicate standard deviations.

### Expression and localization of PcrV in the HeLa cell infection model

HeLa cells (2×10^6^ cells) were seeded in a 60 mm cell culture dish and incubated at 37°C in 5% CO_2_ overnight. HeLa cell monolayers were washed twice with HBSS and incubated in serum-free DMEM for 15 to 20 min. Bacterial cells were suspended in serum-free DMEM and used at an MOI of 5 for *in vitro* infections. After infection for 3 to 4 h, the culture medium containing bacteria was removed and subjected to centrifugation at 5,900× *g* for 10 min to isolate bacterial cells. The proteins in the supernatant were concentrated by ammonium sulfate precipitation for immunoblot analysis. The infected HeLa monolayers were washed with PBS twice and then 500 µl Triton solution (0.05% Triton X-100 and 10 mM EDTA in PBS) was added for fractionation. Cells were scraped and transferred into a microcentrifuge tube. The culture dish was rinsed with additional 100 µl of Triton solution to complete the harvesting of cells. Harvested cells were incubated at room temperature (RT) for 30 min and briefly vortexed several times during the incubation period. The insoluble fractions were collected by centrifugation at 16,100× *g* for 15 min at RT. The supernatant (soluble fractions) represents cellular cytosolic and translocated bacterial proteins.

### Hemolysis assay and erythrocyte protection by Mab166

The hemolysis and protection assays were performed based on modified protocols from Blocker *et. al.* and Goure *et al.* [27], [46]. Sheep red blood cells (RBCs, PML microbiologicals) were washed and stored in 5 mM glucose in PBS. Bacterial cells were grown in LB at 37°C for 3 h, washed and suspended in 5 mM glucose in PBS prior to infection. A mixture of 5×10^7^ RBCs and 2.5×10^7^ bacterial cells (MOI of 0.5) was suspended in the total volume of 200 µl in 0.5 ml microcentrifuge tubes. The samples were subjected to centrifugation at 1,000× g for 10 min at RT and then incubated at 37°C for 1 h. After incubation, RBCs and bacteria were subjected to centrifugation and 150 µl of supernatant was used to assess released hemoglobin as measured by absorbance at 415 nm (SpectraMax M5 by Molecular Devices). To evaluate the protection of RBCs by the monoclonal antibody, 10 µg of MAb166 was incubated with bacterial cells at RT for 45 min and followed by the addition of RBCs for the hemolysis assay. Protection of erythrocytes was calculated by the formula:
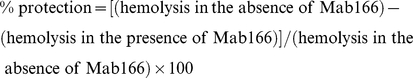
Assays were performed at least 3 times and the error bars indicate standard deviations.

### Surface localization of PcrV derivatives

The flow cytometry procedure used was modified from the method reported by Lee *et al.* [60]. Bacterial cells (initial OD_540_ of 0.1) were grown in LB containing 400 µg/ml carbenicillin in the presence or absence of 2 mM NTA at 37°C for 2.5 h. Cells were collected from 1 ml of culture by centrifugation at 1,300× *g* for 4 min, suspended in 250 µl PBS, and gently mixed by pipetting. For fixation, the same volume of 4% para-formaldehyde in PBS was added and incubated on a rotator for 25 min at room temperature. The reaction was quenched by addition of 25 µl of 1 M Tris-HCl (pH 8) followed by an incubation for 5 min. Fixed cells were washed with PBS and then blocked with 500 µl of 10% fetal bovine serum (FBS) in PBS for 1 h. V-tip protein localization on the bacterial cell surface was measured by assessing the binding of a rabbit anti-PcrV IgG (1∶500) for 1 h. The binding step was followed by two washes with 10% FBS/PBS. Cells were labeled with an Alexa Fluor 488-conjugated anti-rabbit IgG (1∶2,000, Invitogen) for 1 h and washed with 10% FBS/PBS once and then with PBS once. Bacterial cells were subjected to centrifugation and suspended in 750 µl of PBS. The samples were diluted at 1∶200 with PBS prior to flow cytometry analysis using Guava easyCyte 6HT (Millipore). Samples were prepared in duplicate from at least two independent experiments for statistical analyses with SD. For fluorescence microscopy, immunoprobed cells were analyzed with Nikon Eclipse Ti-U inverted microscope with Chroma Sedat filters using a 100× oil immersion objective lens. NIS-Elements software (Nikon) was used for image acquisition. To quantify the surface localization of PcrV, 12 field images were randomly captured for each sample and then the number of bacterial cells possessing the signals of PcrV was compared to the number of DAPI-stained bacterial cells.

## Supporting Information

Figure S1 **Bacterial expression levels of class II and III derivatives with constitutive- secretion phenotype.** Expression of class II and III derivatives and parental PcrV within the *pcrV*-null strain was analyzed after growth in non-inducing (− NTA) and inducing (+ NTA) conditions.
(TIF)
