# Supplementary figures and images for: Modified Needle-Tip PcrV Proteins Reveal Distinct Phenotypes Relevant to the Control of Type III Secretion and Intoxication by Pseudomonas aeruginosa
Source: PLoS One. 2011 Mar 29;6(3):e18356. doi: 10.1371/journal.pone.0018356 (PMC3066235; doi:10.1371/journal.pone.0018356)

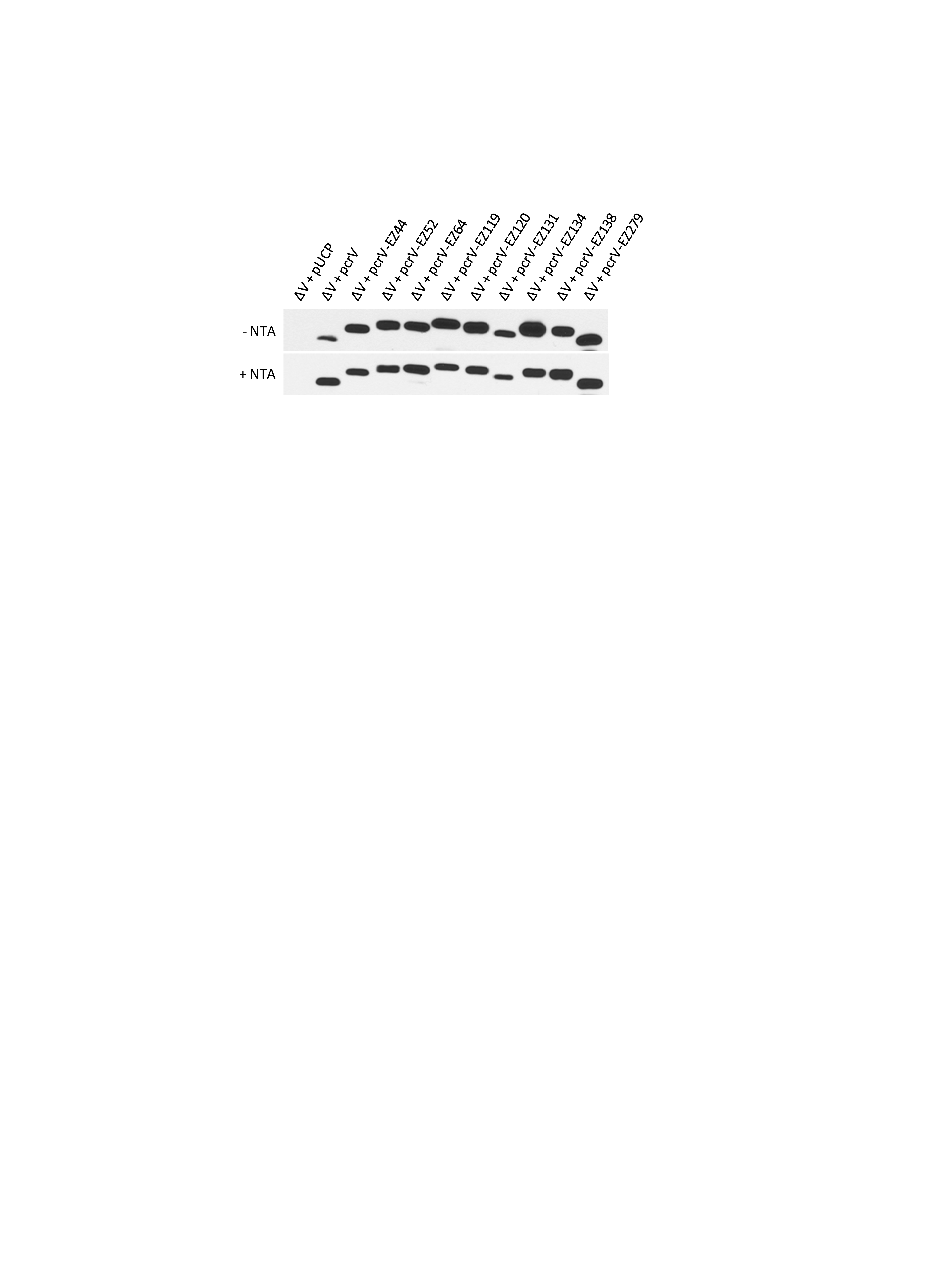

Supplement: Figure S1 — Bacterial expression levels of class II and III derivatives with constitutive- secretion phenotype. Expression of class II and III derivatives and parental PcrV within the pcrV-null strain was analyzed after growth in non-inducing (− NTA) and inducing (+ NTA) conditions. (TIF) [file pone.0018356.s001.tif]
